# Tumor-Intrinsic PD-L1 Promotes Breast Cancer Proliferation Through Livin and Galectin-1-Mediated Regulation of SKP2 Expression

**DOI:** 10.3390/ijms27062741

**Published:** 2026-03-17

**Authors:** Marwa Elfoly, Ayodele Alaiya, Amal A. Al-Hazzani, Monther Al-Alwan, Hazem Ghebeh

**Affiliations:** 1 Innovation and Research, King Faisal Specialist Hospital & Research Centre, MBC 03, P.O. Box 3354, Riyadh 11211, Saudi Arabia; malfoly@kfshrc.edu.sa (M.E.); aalaiya@kfshrc.edu.sa (A.A.); malwan@kfshrc.edu.sa (M.A.-A.); 2 Department of Botany and Microbiology, College of Science, King Saud University, P.O. Box 2460, Riyadh 11451, Saudi Arabia; alhazzani@ksu.edu.sa; 3 College of Medicine, Al-Faisal University, P.O. Box 50927, Riyadh 11533, Saudi Arabia

**Keywords:** PD-L1, proliferation, SKP2, Livin, BIRC7, Galactin-1, LGALS1

## Abstract

Programmed Death-Ligand 1 (PD-L1) promotes tumor progression through several mechanisms, including its intrinsic effect on breast cancer cell proliferation via the S-Phase Kinase-Associated Protein 2 (SKP2)–p21**^Cip1^**/p27**^Kip1^** (SKP2-p21/p27) axis. However, the specific regulatory signaling through which PD-L1 influences the SKP2–p21/p27 axis to drive cell proliferation remains unclear. To investigate how PD-L1 mediates SKP2-dependent proliferation, proteomic analyses, gene-expression manipulation via knockdown or overexpression, Western blotting, quantitative immunofluorescence, colony-forming assays, real-time cell analysis, and Xenograft-derived cells were used. Proteomic data analysis identified several PD-L1 downstream targets as potential candidate regulators of the SKP2–p21/p27 axis and activators of the PI3K/AKT pathway. Candidate screening by gene knockdown, followed by analyses of SKP2, p21, and p27 protein expression, revealed Livin and Galectin-1 as upstream regulators of the SKP2–p21/p27 axis. Moreover, Western blotting and quantitative immunofluorescence in three breast cancer cell lines confirmed that PD-L1 is an upstream regulator of Livin, Galectin-1, and SKP2 protein expression. Mechanistically, Livin and Galectin-1 enhanced AKT phosphorylation (Ser473) to sustain PI3K/AKT pathway activation in a positive feedback loop to upregulate SKP2 expression. Functional assays, including colony-forming assays and real-time cell analyzer, demonstrated that Livin and Galectin-1 are critical for PD-L1-mediated, SKP2-dependent proliferation. These findings were corroborated in vivo using xenograft-derived cells. Overall, these findings delineate a tumor-intrinsic signaling axis in which PD-L1 upregulates Livin and Galectin-1 to sustain PI3K/AKT activity and drive SKP2-dependent cell proliferation. Targeting Livin and/or Galectin-1 may provide a rational strategy to disrupt PD-L1-associated proliferative signaling and improve combinatorial therapeutic approaches in breast cancer.

## 1. Introduction

Breast cancer (BC) is a leading cause of cancer-related deaths in women [1]. Uncontrolled proliferation, a hallmark of cancer, is driven by dysregulation of cell division pathways, such as PI3K/AKT, MAPK/ERK, and Wnt/β-catenin [2,3]. While standard treatment involves chemotherapy, which typically targets rapidly dividing cancer cells, such therapy becomes less effective in advanced stages, particularly in metastatic disease. Alongside chemotherapy, the immune checkpoint inhibitors, anti-PD-1/PD-L1 agents, may also be indicated to enhance treatment efficacy in triple-negative breast cancer (TNBC), a subtype of breast cancer characterized by the absence of estrogen and progesterone receptors and the lack of HER2/neu amplification.

Although PD-L1 plays a major role in immune evasion, it also has an immune-independent intrinsic tumor-promoting effect [4]. Evidence suggests that this intrinsic function of PD-L1 contributes to resistance to checkpoint inhibitors by activating AKT and/or ERK pathways [5]. Insights from dissecting PD-L1’s intrinsic role could inform the design of optimized combination therapies to overcome resistance.

Our previous work, along with others, identified PD-L1’s intrinsic role in promoting BC cell stemness and self-renewal via the PI3K/AKT pathway [6,7]. Additionally, we demonstrated that PD-L1 drives proliferation of BC cells via the SKP2–p21/p27 axis [8], which is consistent with our earlier findings in BC patients correlating PD-L1 with proliferation markers, such as Ki-67 and the mitotic index [9]. However, the mechanism by which PD-L1 regulates the SKP2–p21/p27 axis remains unclear.

Livin, encoded by Baculoviral IAP Repeat-Containing 7 (BIRC7), is predominantly expressed in cancer cells, with minor, if any, expression in normal differentiated cells [10]. Livin expression correlates with worse prognoses in many types of cancer [11]. We previously showed that Livin is upregulated in response to chemotherapy [12], which is consistent with its reported role in tumor cell resistance to many chemotherapeutic agents (reviewed by Liu et al. [13]). Similarly, Galectin-1, encoded by LGALS1, is involved in many cellular functions, including invasion [14] and immune evasion [15,16], and is associated with the PI3K/AKT pathway [17]. There is evidence that Galectin-1 induces resistance to chemotherapy [18,19,20].

In this study, we used TNBC cells to present evidence showing that PD-L1 upregulates SKP2 expression through the PI3K/AKT pathway-associated molecules Livin and Galectin-1. Consequently, SKP2 modulates the p21 and p27 expression to drive the cell cycle progression.

## 2. Results

We previously reported that PD-L1-expressing xenografts exhibited significantly larger tumors than their PD-L1 knockdown (PD-L1^KD^) counterparts [7]. In a subsequent study, we demonstrated that PD-L1 promotes cell cycle progression by upregulating SKP2, which, in turn, modulates p21 and p27 to drive cell cycle progression [8]. In the present study, we investigated how PD-L1 regulates the SKP2–p21/p27 axis.

### 2.1. Livin and Galectin-1 Modulate the Expression of SKP2–p21/p27

We hypothesized that PD-L1 signaling converges on nuclear regulators that drive the transcription of proliferation-associated genes [8]. Moreover, nuclear PD-L1 (nPD-L1) could directly interact with proliferation-related proteins within the nucleus. To investigate how PD-L1 regulates the SKP2–p21/p27 axis, we analyzed our previously generated nuclear proteomics data of differentially expressed proteins in PD-L1-expressing (PD-L1^Cont^) vs. PD-L1 knockdown (PD-L1^KD^) MDA-MB-231 TNBC cells (Supplementary Dataset File S1) [8].

**Figure 1:**
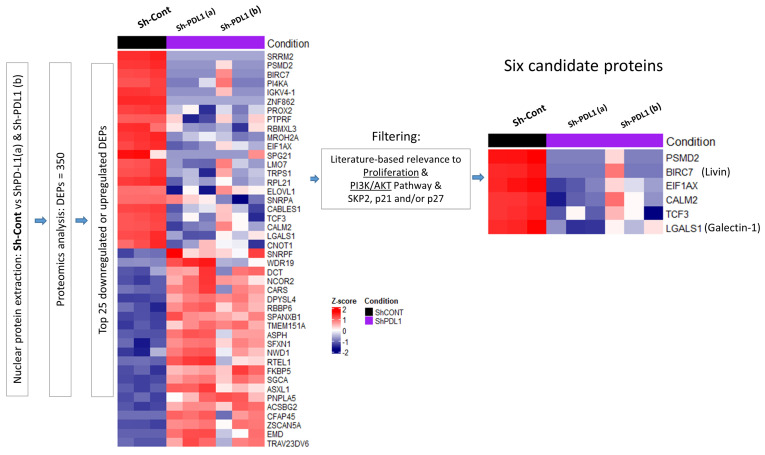
**Proteomic analysis of nuclear extracts from PD-L1^Cont^ (Sh-Cont) vs. PD-L1^KD^ (ShPD-L1(a) and Sh-PDL1(b)) clones of MDA-MB-231 breast cancer cells.** Schematic overview of the proteomics-guided workflow used to identify PD-L1-dependent regulators of the SKP2/p21/p27 axis. The heatmap displays the top nuclear differentially expressed proteins (DEPs) in Sh-Cont compared to Sh-PD-L1(a) and Sh-PD-L1(b) (|log_2_FC| ≥ 2 and adjusted *p*-value ≤ 0.05). DEPs were further filtered based on literature-based relevance to proliferation, the PI3K/AKT pathway, and SKP2, p21, and/or p27 expression (details are in Supplementary Table S3 and Supplementary Data File S1).

A heatmap displaying the top 25 nuclear proteins was generated from the 350 differentially expressed proteins (DEPs) identified in PD-L1 knockdown cells (Sh-PD-L1 a/b) versus control cells (Sh-Cont) (Figure 1), including those downregulated (top) and upregulated (bottom). Because proteins reduced in PD-L1 knockdown cells are enriched in PD-L1-expressing cells, this enriched set was literature-filtered for their roles in proliferation, PI3K/AKT activation, and regulation of SKP2, p21, or p27 (Figure 1, see Supplementary Table S3 for details), yielding six candidates: PSMD2, Livin (BIRC7), EIF1AX, CALM2, TCF3, and Galectin-1 (LGALS1).

**Figure 2:**
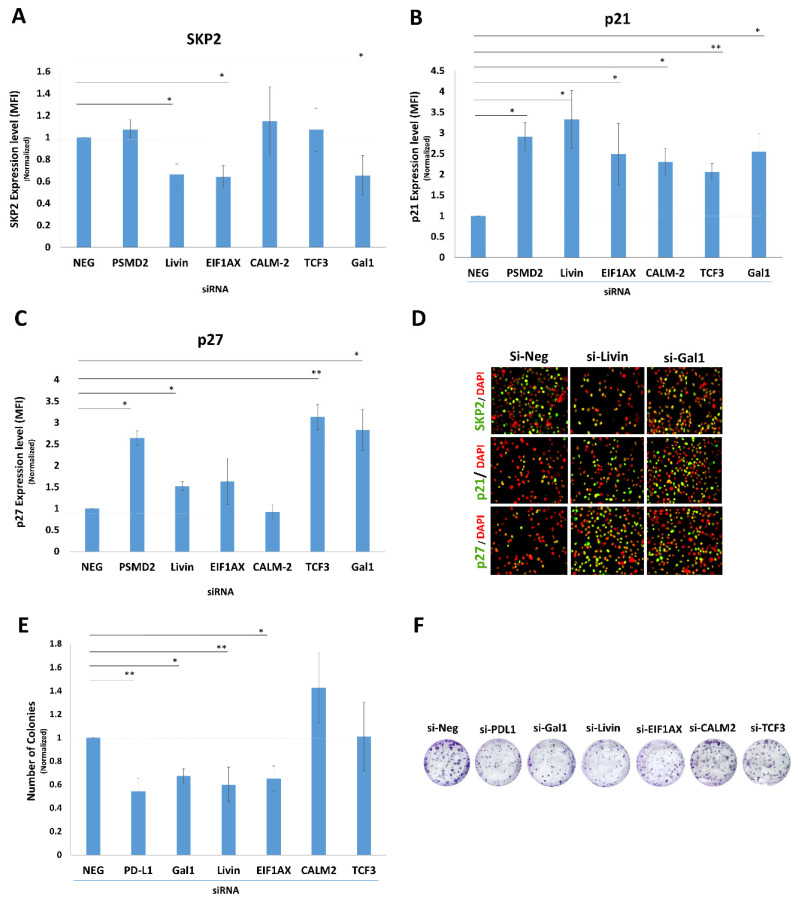
**Knockdown of Livin and Galectin-1 downregulates SKP2 expression and inhibits colony formation.** Expression levels of SKP2 (**A**), p21 (**B**), and p27 (**C**) in MDA-MB-231 cells following transient knockdown of six candidate upstream proteins measured using qIF. Data were normalized to negative control siRNA (si-Neg) and are presented as means ± SEMs from four independent experiments. (**D**) Representative IF images (×200 magnification) showing SKP2, p21, and p27 expression after Livin or Galectin-1 knockdown compared with control siRNA. (**E**) Colony-forming ability of MDA-MB-231 cells after transient knockdown of candidate genes. Data were normalized to negative control siRNA–treated cells and are displayed as means ± SEMs from three independent experiments (*n* = 3). (**F**) Representative colony-forming assays following knockdown of PD-L1, Livin, Galectin-1, EIF1AX, CALM2, and TCF-3 compared to control siRNA. Statistical significance is indicated as follows: * *p* < 0.05, ** *p* < 0.01.

To screen the six proteomics-nominated candidates and relate them to PD-L1-mediated regulation of the SKP2–p21/p27 axis and cell proliferation, each gene was individually silenced in MDA-MB-231 TNBC cells and evaluated using two complementary readouts: (i) modulation of the SKP2–p21/p27 axis and (ii) colony-forming ability (CFA) as a measure of their proliferative capacity. Among the knocked-down candidates, Livin, EIF1AX, or Galectin-1 significantly reduced SKP2 expression as shown by quantitative immunofluorescence (qIF) (Figure 2A). In contrast, knockdown of any of the six candidate genes significantly increased p21 expression (Figure 2B), whereas only PSMD2, Livin, TCF3, or Galectin-1 knockdown significantly increased p27 expression (Figure 2C). Of the six proteomics-nominated candidates, only Livin and Galectin-1 knockdown resulted in concurrent SKP2 downregulation together with upregulation of both p21 and p27 (Figure 2D). Consistent with these molecular effects, CFA was significantly inhibited by the knockdown of Livin, Galectin-1, and EIF1AX to an extent comparable to PD-L1 knockdown (Figure 2E,F). Although EIF1AX knockdown reduced SKP2 and inhibited CFA, it did not significantly increase p27. Integrating the molecular (SKP2-p21/p27) and functional (CFA) readouts, Livin and Galectin-1 emerged as the most consistent candidates, demonstrating concurrent SKP2 downregulation with p21/p27 upregulation together with reduced proliferative capacity, and were therefore prioritized for subsequent mechanistic analyses.

### 2.2. PD-L1 Is an Upstream Regulator of Livin and Galectin-1 Expression in TNBC

**Figure 3:**
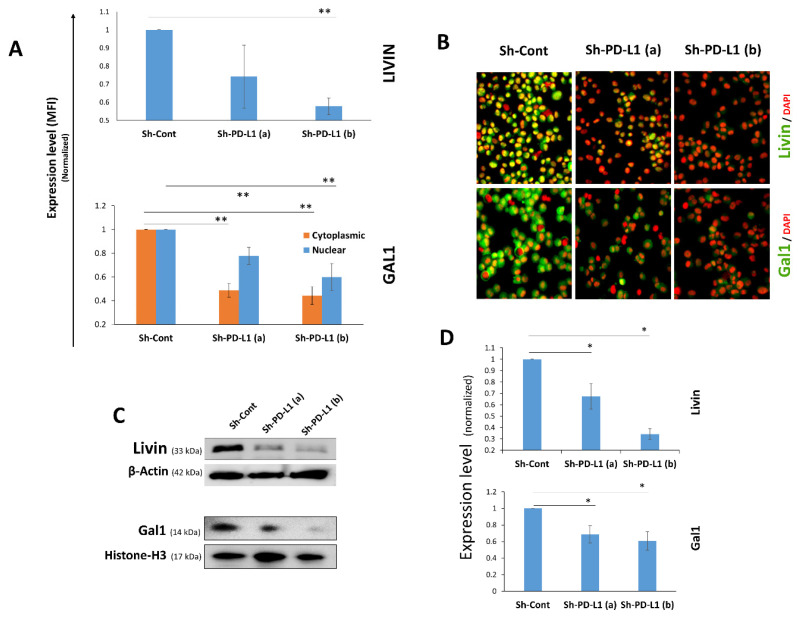
**PD-L1 promotes Livin and Galectin-1 expression in TNBC cells.** (**A**) Livin and Galectin-1 expression levels in PD-L1^KD^ MDA-MB-231 clones compared with PD-L1^Cont^ controls, measured using qIF. Data were normalized to Sh-Cont MFI and are presented as means ± SEMs from four independent experiments. (**B**) Representative IF images of PD-L1^KD^ clones and controls (×200 magnification). (**C**) Western blot analysis of Livin and Galectin-1 expression following PD-L1 knockdown in MDA-MB-231 cells. (**D**) Densitometric quantification of the Western blots from three independent experiments presented as mean ± SEM (*n* = 3). Statistical significance is indicated as follows: * *p* < 0.05, ** *p* < 0.01.

The impact of PD-L1 on Livin or Galectin-1 expression was assessed via qIF and Western blotting. Livin, which is predominantly localized in the nucleus, showed significantly reduced expression in PD-L1^KD^ cells compared with PD-L1^Cont^ cells, as shown by qIF (Figure 3A,B). Consistent with the qIF results, Livin was significantly downregulated in both PD-L1^KD^ clones, as shown by the Western blot (Figure 3C,D). Galectin-1, which is expressed in both nuclear and cytoplasmic fractions, was significantly suppressed in both compartments in PD-L1^KD^ cells compared with their PD-L1^Cont^ counterparts (Figure 3A,B). Western blot analysis of nuclear Galectin-1, the protein fraction that is closely linked to proliferation [21], confirmed the qIF findings and revealed significantly lower expression in PD-L1^KD^ cells compared with PD-L1^Cont^ cells (Figure 3C). Furthermore, transient PD-L1 knockdown in MDA-MB-231 cells reduced both Galectin-1 and Livin expression levels (Supplementary Figure S2), thus ruling out the off-target effects of PD-L1 ShRNA in PD-L1^KD^ cells. These findings were further confirmed in SUM159, another TNBC cell line. Livin was downregulated in both stable PD-L1 knockdown (PD-L1^KD^) SUM159 clones, while Galectin-1 was downregulated in one clone, as shown by both qIF and Western blot (Supplementary Figure S3).

**Figure 4:**
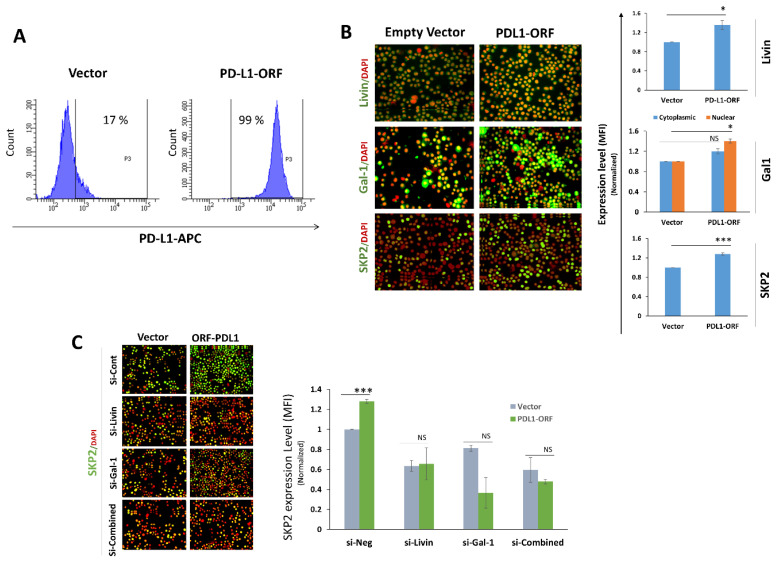
**PD-L1 overexpression in MDA-MB-468 breast cancer cells upregulates SKP2 in a Livin/Galectin-1-dependent manner.** (**A**) PD-L1 expression measured by flow cytometry following stable overexpression of PD-L1 in MDA-MB-468 cells using a PD-L1 ORF construct (PD-L1-ORF), compared with empty vector-transfected cells (vector control). (**B**) Expression of Livin, Galectin-1, and SKP2 in stable PD-L1 ORF MDA-MB-468 cells, measured using qIF. (**C**) SKP2 expression upon transient knockdown of Livin, Galectin-1, or their combination (combined) in PD-L1-ORF and vector control MDA-MB-468 cells. Data were normalized to empty vector cells and are shown as means ± SEMs from three independent experiments (right) (*n* = 3). Representative IF images (×200 magnification) (left). Statistical significance is indicated as follows: * *p* < 0.05, *** *p* < 0.001, NS, not significant.

To validate the effect of PD-L1 on Livin/Galectin-1/SKP2, we overexpressed PD-L1 using PD-L1 ORF in MDA-MB-468, a TNBC cell line that naturally exhibits low or negligible PD-L1 expression [22], making it suitable for PD-L1 overexpression (Figure 4A). Overexpression of PD-L1 (PDL1-ORF) in these cells significantly upregulated Livin, Galectin-1 and SKP-2 expression as compared to the empty vector (vector) transfected control cells (Figure 4B). Importantly, knocking down Livin, Galectin-1, or their combination, abrogated the increased SKP2 expression in PD-L1 ORF MDA-MB-468 cells as compared to the empty vector control cells (Figure 4C). Interestingly, knockdown of Livin or Galectin-1 reduced SKP2 expression even in vector control cells, supporting a PD-L1-independent role for Livin and Galectin-1 in regulating SKP2 expression.

### 2.3. PD-L1 Upregulates Livin and Galectin-1 Expression to Promote SKP2-Dependent Cell Proliferation

**Figure 5:**
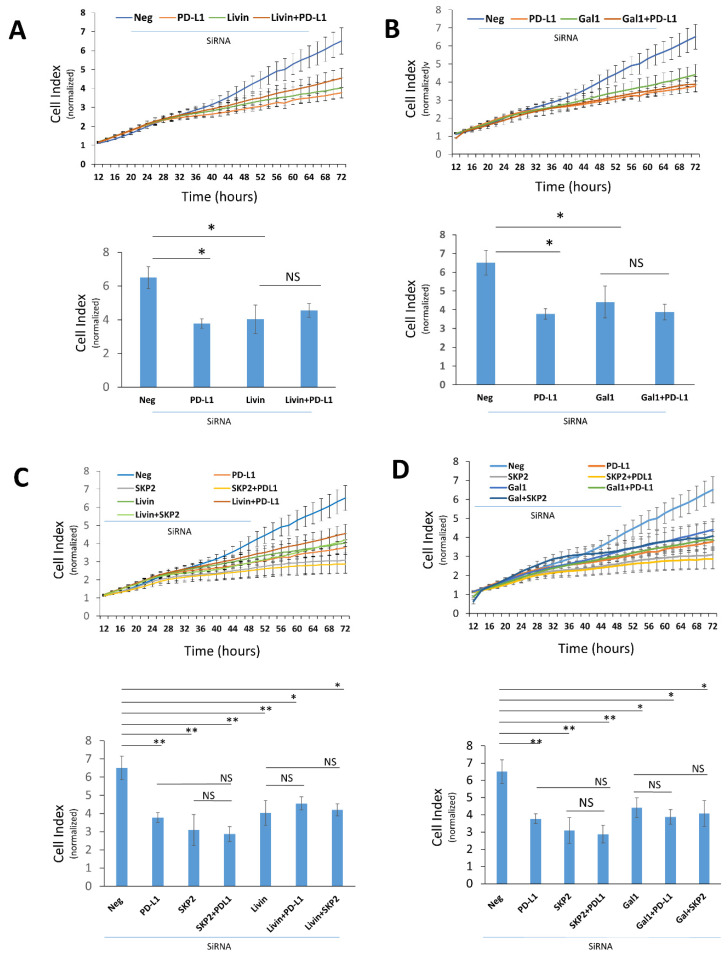
**Knockdown of SKP2, Livin, or Galectin-1 abrogates PD-L1-promoted TNBC cell proliferation.** MDA-MB-231 breast cancer cell proliferation was monitored in real time using the RTCA system. Cell index values are shown over 72 h (**upper panel**, line graph) and at the 72 h endpoint (**lower panels**, bar graphs; *n* = 3). Cells were transfected with siRNA targeting PD-L1 alone or in combination with Livin (**A**) or Galectin-1 (**B**), with or without SKP2 (**C**,**D**), and were compared with scrambled siRNA controls. Data are presented as mean ± SEM from at least three independent experiments (*n* = 3). Statistical significance is indicated as follows: * *p* < 0.05, ** *p* < 0.01; NS, not significant.

To assess the functional relevance of PD-L1-mediated Livin and Galectin-1 expression and their relationship to SKP2, we tested the effect of single and combined gene silencing on MDA-MB-231 cell proliferation using RTCA. Knockdown of Livin or Galectin-1 inhibited cell proliferation to a degree comparable to PD-L1 silencing (Figure 5A,B). Importantly, simultaneous knockdown of Livin and PD-L1 or Galectin-1 and PD-L1 did not produce a greater inhibition of cell proliferation than single-gene silencing (Figure 5C,D), indicating that Livin or Galectin-1 knockdown abrogates the PD-L1 effect on cell proliferation. Similarly, combined silencing of Livin and SKP2 or Galectin-1 and SKP2 did not result in an additional inhibition of cell proliferation compared with single-gene knockdown, stressing on the role of Livin and Galectin in the SKP2-depdenent effect of PD-L1 on cell proliferation (Figure 5C,D). Taken together, our data demonstrate that PD-L1-mediated Livin and Galectin-1 expression is important for the SKP2-dependent effect on PD-L1 cell proliferation.

### 2.4. Positive Feedback Loop Involving Livin/Galectin-1 and PI3K/AKT Activation

**Figure 6:**
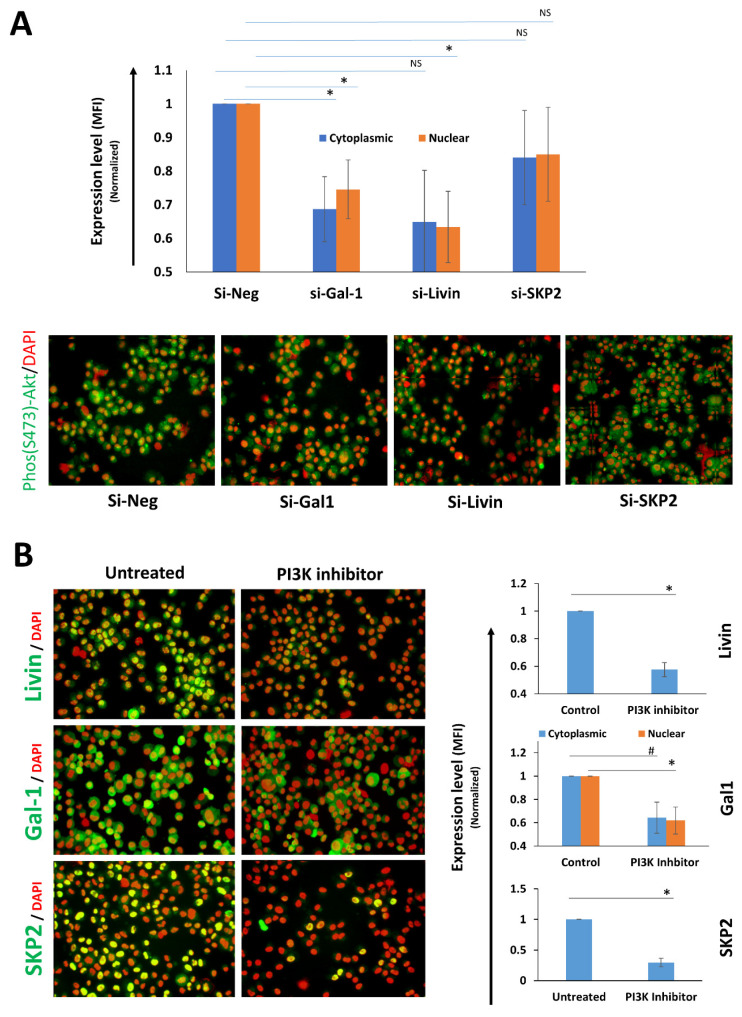
**Livin and Galectin-1 promote PI3K/AKT pathway activation and vice versa.** (**A**) Top: quantitative immunofluorescence (qIF) showing MFI of p-AKT (S473) in MDA-MB-231 cells upon transient knockdown of Livin, Galectin-1, or SKP2, normalized to si-Neg and presented as means ± SEMs from three different experiments. Bottom: representative IF images (×200 magnification). (**B**) Left: representative IF images (×200 magnification) showing Livin, Galectin-1, and SKP2 expression after PI3K inhibition with LY294002 treatment. Right: bar graph with normalized qIF data shown as means ± SEMs from three independent experiments (*n* = 3). (*, # and NS) indicates statistical significance (* statistically significant *p* < 0.05, # = borderline significance, NS = not significant).

To understand how Livin and Galectin-1 regulate SKP2 expression, we checked their effect on the PI3K/AKT pathway, a critical pathway for SKP2 expression regulation [23] that also crosstalks with Livin and Galectin-1 [17,24]. To assess the relationship of Livin/Galectin-1 to the PI3K/AKT pathway, we examined AKT phosphorylation following Livin, Galectin-1, and SKP2 knockdown in MDA-MB-231 cells. Transient knockdown of either Livin or Galectin-1 significantly decreased AKT phosphorylation in the nuclear fraction, while a significant reduction in the cytoplasmic fraction of p-AKT was observed only in the Galectin-1 knockdown group (Figure 6A). In contrast, knockdown of SKP2 did not significantly affect AKT phosphorylation. Reciprocally, inhibition of the PI3K/AKT pathway using LY294002 significantly reduced Livin, Galectin-1, and SKP2 expression (Figure 6B), suggesting the presence of a positive feedback loop between Livin/Galectin-1 and PI3K/AKT pathway activation that is upstream of SKP2.

### 2.5. PD-L1 Expression Correlates with SKP2–p21/p27 and Livin/Galectin-1 Expression In Vivo

To extend our analysis to in vivo human data, we initially examined correlations among the studied molecules and proliferation using publicly available datasets. Analysis of TCGA BC microarray data revealed a significant positive correlation between PD-L1, Livin, Galectin-1, or SKP2 with the proliferation signature score (Supplementary Figure S4).

**Figure 7:**
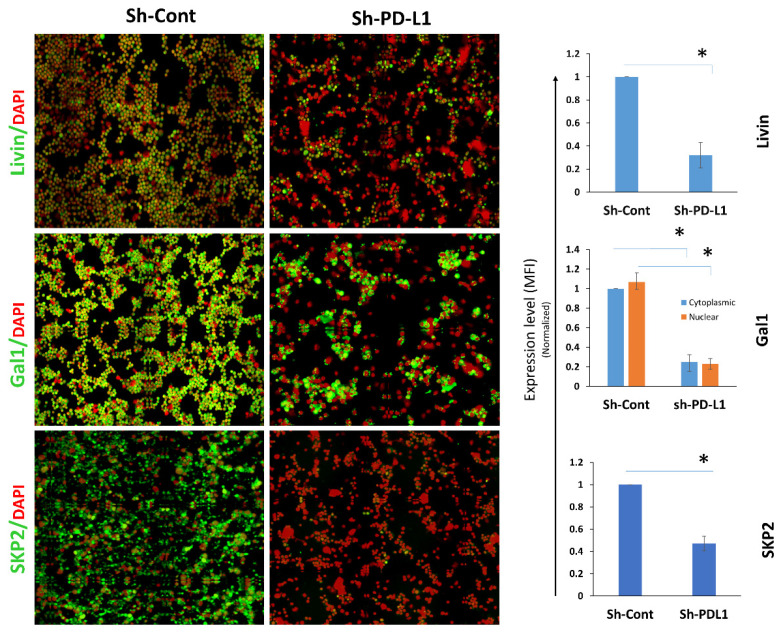
**PD-L1 modulates Livin and Galectin-1 expression in vivo.** Expression of Livin and Galectin-1 in PD-L1^Cont^ and PD-L1^KD^ MDA-MB-231 xenografts. (**Left**) Representative IF images (×100 magnification). (**Right**) Bar graphs showing expression levels (mean ± SEM), measured using qIF, normalized to Sh-Cont from three different experiments (*n* = 3). (*) indicates statistical significance (*p* < 0.05).

To further validate the effect of PD-L1 on Livin, Galectin-1, and SKP2 in vivo, we analyzed cryopreserved single cells previously isolated from xenografts derived from PD-L1^KD^ cells and their control counterparts, as previously reported [7]. Our qIF analysis revealed significant downregulation of Livin, Galectin-1, and SKP2 levels in PD-L1^KD^ compared with PD-L1^Cont^ cells (Figure 7).

**Figure 8:**
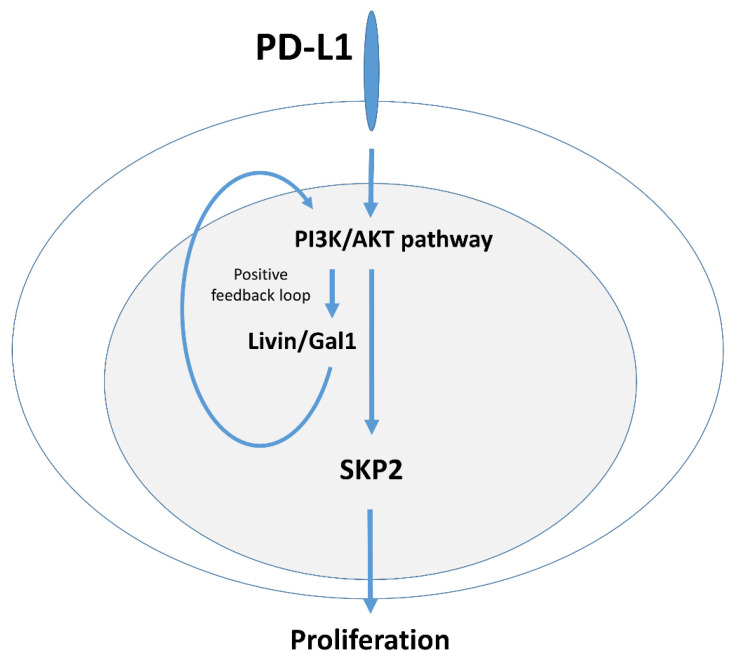
**PD-L1 upregulates Livin/Galectin-1 expression, activating the PI3K/AKT pathway to promote proliferation through SKP2–p21/p27.** Schematic illustration of PD-L1-mediated PI3K/AKT activation via Livin and Galectin-1, which sustains PI3K/AKT signaling through a positive feedback loop. This PI3K/AKT activation upregulates SKP-2 and downregulates p21/p27 expression to drive cell cycle progression. Arrows indicate a promoting effect.

Our data demonstrate that PD-L1 upregulates Livin and Galectin-1 expression via the PI3K/AKT pathway, which in turn activates the PI3K/AKT pathway further as shown by phosphorylated AKT(s473), forming a putative positive feed loop. This cascade leads to SKP2 upregulation, which modulates p27 and p21 expression, thereby promoting cell cycle progression and cell proliferation (Figure 8).

## 3. Discussion

PD-L1 is a known promoter of breast cancer (BC) cell proliferation [8,25,26,27]. Our previous work identified the SKP2–p21/p27 axis as a key mediator of PD-L1-induced proliferation. However, the upstream signaling regulating SKP2 remained unclear. This study demonstrates for the first time that PD-L1 modulates the expression of Livin and Galectin-1, which, in turn, regulate the SKP2–p21/p27 axis to drive BC cell proliferation. While proteomic analysis identified several nuclear proteins, three of which (Livin, EIF1AX, and Galectin-1) inhibited proliferation in CFA assays (Figure 2F), only Livin and Galectin-1 modulated the SKP2–p21/p27 axis.

The PI3K/AKT pathway is a large signaling network that integrates signals across multiple cellular processes, including cell proliferation, growth, metabolism, and autophagy. The coordination of these processes requires flexible, robust, and tight control mechanisms. Thus, positive feedback or feed-forward loops are plausible mechanisms that serve to amplify weak signals, stabilize responses, and promote rapid activation. Accordingly, subtle changes and a limited number of protein–protein interactions may result in strong signals (reviewed in [28]). In contrast, negative feedback loops can limit the overactivation of pathways under different conditions.

There is ample evidence that the intrinsic PD-L1 expression can activate the PI3K/AKT pathway [7,27,29,30]. In turn, AKT stabilizes the SKP2 protein through phosphorylation at Ser72 [31], or indirectly via mTOR-mediated phosphorylation at Ser64 [32]. However, activation of the PI3K pathway is typically transient [33], as the signal rapidly diminishes unless sustained by continuous upstream activation. In the present study, we demonstrate that Livin and Galectin-1 sustain PI3K/AKT pathway activation through a positive feedback loop, thereby increasing SKP2 levels and promoting proliferation (Figure 8).

Livin, a member of the inhibitor of apoptosis family, is overexpressed in BC, among other cancers [24,34], but is rarely detectable in normal breast tissues [35]. Consistent with previous findings indicating that Livin promotes BC cell proliferation through PI3K/AKT pathway activation and its knockdown induces G0/G1 cell cycle arrest [24], our study shows that Livin drives SKP2-mediated cell cycle progression. This aligns with our prior findings showing that PD-L1 knockdown arrests the cell cycle in the G0/G1 phase [8]. Indeed, TCGA BC data analysis corroborated a significant positive correlation of Livin and SKP2 with the proliferation signature score, which comprises 512 proliferation-related genes. Our findings of reduced AKT phosphorylation when Livin was knocked down is consistent with previous studies showing that Livin promotes PI3K/AKT activation in a similar manner to PD-L1 [7,24]. Furthermore, inhibition of the PI3K/AKT pathway reduced Livin expression, supporting the existence of a positive feedback loop involving Livin and the PI3K/AKT pathway.

Galectin-1, a member of the carbohydrate-binding lectin family, is localized in the nucleus and cytoplasm, with nuclear Galectin-1 being critical for mammary cell proliferation [21,36]. Additionally, Galectin-1 is involved in various cellular functions, including invasion [14] and immune evasion [15,16]. OTX008, a selective Galectin-1 inhibitor, suppresses tumor cell proliferation and invasion by inhibiting AKT and ERK phosphorylation [37]. In the present study, Galectin-1 knockdown in BC cells upregulated p27 and p21 expression and inhibited cell proliferation, consistent with findings in oral cancer cells where Galectin-1 knockdown upregulated p27 expression and arrested the cell cycle in the G0/G1 phase [38]. Consistent with our results in BC cells, Galectin-1 was also reported to regulate the PI3K/AKT/mTOR pathway, as its knockdown reduces AKT and mTOR phosphorylation in urothelial cancer cells [17]. Conversely, Galectin-1 can function downstream of PI3K/AKT, and inhibiting PI3K was shown to reduce Galectin-1 expression in ovarian cancer cells [39]. Similar to Livin, our data suggest that Galectin-1 participates in a positive feedback loop within the PI3K/AKT pathway, promoting SKP2–p21/p27 axis–mediated cell cycle progression and proliferation in BC cells.

Combining PI3K/AKT pathway inhibitors with PD-L1 blockade is under investigation in clinical trials [40]. Therefore, a better understanding of PD-L1-induced PI3K/AKT activation and its effects on cell proliferation can inform better therapeutic strategies, including PD-L1 inhibitor design and PD-L1–PI3K/AKT pathway inhibitor combinations. In this study, we demonstrated that PD-L1^KD^ downregulates PI3K/AKT-associated signaling proteins, Livin and Galectin-1, thereby inhibiting SKP2-dependent BC cell proliferation. Notably, previous studies have reported SKP2-mediated resistance to PI3K/AKT pathway inhibitors [41]. Therefore, targeting SKP2, or alternatively—as suggested by the present findings—Livin or Galectin-1, may represent a potential strategy to overcome resistance associated with PI3K/AKT inhibitors. However, a limitation of this study is that it was restricted to TNBC cell lines and experimental in vitro and in vivo models. Validation of these findings in patient tumor samples in future studies will be important.

## 4. Methods and Materials

### 4.1. Cell Culture and Treatment

The MDA-MB-231 cell line (cellosaurus:CVCL_0062), obtained from ATCC (Manassas, VA, USA), was maintained in high-glucose DMEM supplemented with 10% fetal bovine serum (FBS) (Sigma, St. Louis, MO, USA). MDA-MB-468 cell line (cellosaurus:CVCL_0419), obtained from ATCC, was maintained in DMEM/F12 supplemented with 10% FBS. SUM159PT (cellosaurus:CVCL_5423) cells, obtained from Asterand (Detroit, MI, USA), were cultured in DMEM/F12 supplemented with insulin, hydrocortisone, and 5% FBS as previously described [7,8].

Protein expression was assessed during the exponential growth phase (24–48 h), as reported previously [7,8]. To limit feedback effects in the PI3K/AKT pathway [42], cells were serum-starved for 24–30 h before culturing in complete medium for 18–24 h to analyze Livin and Galectin-1 protein expression. For PI3K/AKT pathway inhibition experiments, cells were treated with LY294002 at 20µM concentration for 24 h prior to downstream expression analysis. Experiments were repeated at least 3 times to generate data.

### 4.2. Gene Expression Knockdown

The PD-L1 knockdown (PD-L1^KD^) stable clones, sh-PD-L1(a) and sh-PD-L1(b), along with the scrambled shRNA control (Sh-Cont) of MDA-MB-231 cells, were used as previously described [7,8]. PD-L1^KD^ SUM159PT clones were also previously reported [8] (Supplementary Figure S1A,B). Transient gene knockdown was performed using siRNA and Lipofectamine RNAiMAX (Thermo Fisher Scientific, Waltham, MA, USA) following the manufacturer’s instructions, as previously reported [43]. Custom siRNAs were designed using the Kay Lab siRNA/shRNA/Oligo Optimal Design website (http://web.stanford.edu/group/markkaylab/cgi-bin/ accessed on 18 March 2020) and synthesized by Metabion International AG (Planegg, Germany) (Supplementary Table S1).

### 4.3. PD-L1 ORF Overexpression

PD-L1 ORF (RC213071 from OriGene, Rockville, MD, USA) was overexpressed in MDA-MB-468 cells using Lipofectamine LTX (Thermo Fisher Scientific), followed by selection using the eukaryotic antibiotic, G 418 (Thermo Fisher Scientific) at 750 µg/mL.

### 4.4. Colony-Forming Assay

Cells (500 per well) were seeded into 6-well plates with complete DMEM. After 14 days, colonies were washed with PBS, fixed with 4% formaldehyde, stained with crystal violet, and counted, following a previously described protocol [8,44].

### 4.5. Tissue Processing of Xenograft Tissues

Cryopreserved single cells in a 10% DMSO-based freezing medium were prepared by mincing xenograft tumors and digesting with collagenase (Stem Cell Technologies, Vancouver, Canada) under agitation at 37 °C for one hour. The xenograft tumors were previously generated in our published animal work (Almozyan, S. et al. [7]) of nude (obtained from Jackson Labs, Bar Harbor, ME, USA) mouse xenograft tumors of PD-L1^KD^ MDA-MB-231 cells (Sh-PD-L1(a)) or their control (Sh-Cont), as described in detail. [7].

### 4.6. Cell Proliferation Assays

Cell proliferation was monitored using the xCELLigence Real-Time Cell Analyzer (RTCA) system (ACEA Biosciences Inc., San Diego, CA, USA). Cells (20,000 per well) were seeded into E-plates with 200 µL of medium, and cell number changes were calculated using RTCA Software Package 2.0, as previously described [8].

### 4.7. Western Blotting

Proteins were separated by SDS-PAGE and transferred to polyvinylidene fluoride membranes. The membranes were incubated with primary antibodies (Supplementary Table S2) diluted in Tris-buffered saline with Tween 20, per the manufacturer’s guidelines, followed by secondary antibodies. Signals were developed using the SuperSignal Kit (Thermo Fisher Scientific, Waltham, MA, USA) as previously reported [8]. The membrane images were captured using the ImageQuant LAS 4000 system (GE Healthcare, Uppsala, Sweden) with its associated software and automatic exposure settings. Images from at least 3 independent experiments were quantified using ImageJ software, version 1.54r and presented as mean ± SEM, unless otherwise indicated. All adjacent bands were cropped from the same gel, and if necessary, the whole image was adjusted for brightness/contrast. Protein loading was assessed using GAPDH, β-actin, or Histone H3, either on the same membrane as the protein of interest, or on a separate membrane loaded with identical protein content. Cytoplasmic and nuclear protein fractionation was done using the NE-PER™ reagent (Thermo Fisher Scientific) and validated using GAPDH and histone H3, respectively, as previously described [45].

### 4.8. Quantitative Immunofluorescence (qIF)

Cells were cytospun, air-dried (3–24 h), and immunostained following Cell Signaling Technology protocols. Primary antibodies are listed in Supplementary Table S2. Fluorescence images were captured using the BD Pathway 855 image analyzer, and intensities were quantified via BD AttoVision image acquisition software, version 1.5 (both BD Biosciences, Franklin Lakes, NJ, USA). As the camera captures images in black and white, pseudocolors were applied for visualization (red for DAPI and green for Alexa Fluor 594). Mean fluorescence intensity (MFI) from the cytoplasmic and nuclear regions was measured following image segmentation based on DAPI staining of the nuclear region. Data acquisition was carried out using the 3 × 3 montage mode of the AttoVision software, with around 2000 cells analyzed per montaged image. Final analysis and calculation of mean fluorescence intensity (MFI) for protein expression were performed using BD Image Data Explorer software, version 2.2.15 (BD Biosciences), based on at least three independent experiments [7].

### 4.9. Proteomics/Bioinformatics Analysis

Proteomics data previously reported [8] were further analyzed, where protein intensity values from the nuclear fractions of Sh-Cont and Sh-PDL1 ((a) and (b)) samples were log_2_-transformed to stabilize variance and reduce skewness before analysis. Proteomics analysis was performed on data obtained from three biological replicates for each condition. Differential analysis was performed using the limma R-package, version 3.64.3. Proteins with an absolute log_2_ fold change (log_2_FC) of at least 0.5 and an adjusted *p*-value ≤ 0.05 (Benjamini–Hochberg correction) were considered significantly expressed. Proteomics data visualization was performed using the ComplexHeatmap, version 2.24.1.

For patients’ data analysis, the publicly available dataset (The Cancer Genome Atlas (TCGA) BC Nature 2012) was used. Correlations and statistics were obtained directly from this platform. The dataset was further downloaded and analyzed to assess the correlation between the proliferation signature (MSigDB, GO:0008283; C5: GO Biological Process, 512 genes) and PD-L1, LGALS1, and BIRC7. The gene signature score was calculated as the average expression of all genes in each signature across all patient samples. Correlations between gene expression and signature scores were evaluated using Spearman’s rank correlation.

### 4.10. Statistical Analysis

All results were normalized to untreated/control cells and expressed as means ± standard errors of means (SEMs). Statistical significance between groups was determined using the paired Student’s *t*-test, with *p* < 0.05 indicating statistical significance.

## 5. Conclusions

In this study, we demonstrate that tumor-intrinsic PD-L1 promotes breast cancer cell proliferation through a previously unrecognized regulatory mechanism involving Livin and Galectin-1, which links PD-L1 signaling to SKP2 regulation. PD-L1 functions as an upstream regulator of Livin and Galectin-1 expression, leading to enhanced AKT phosphorylation at Ser473. In turn, activation of the PI3K/AKT pathway further upregulated Livin and Galectin-1 expression, establishing a positive feedback loop that sustains PI3K/AKT signaling. Persistent PI3K/AKT activation subsequently upregulates SKP2 expression, driving cell proliferation through the SKP2–p21/p27 axis.

Collectively, these findings expand our understanding of PD-L1 biology beyond its role in immune evasion. The identification of Livin and Galectin-1 as key intermediates provides mechanistic insight into PD-L1-associated oncogenic signaling and highlights potential therapeutic targets. Targeting Livin and/or Galectin-1 in combination with PD-L1 blockade may represent a more effective strategy, while potentially avoiding the systemic toxicities associated with direct PI3K inhibition.

## Data Availability

The original contributions presented in this study are included in the article/Supplementary Materials. Further inquiries can be directed to the corresponding author.

## Supplementary Materials

The following supporting information can be downloaded at: https://www.mdpi.com/article/10.3390/ijms27062741/s1.

## Abbreviations

BC: Breast Cancer, PD-L1^KD^: PD-L1 knockdown, scrambled shRNA control (Sh-Cont), RTCA: real-time cell analyzer, CFA: colony forming ability, qIF: quantitative immunofluorescence; TCGA: The Cancer Genome Atlas. Gal1: Galectin-1
