# Supplementary material for: Tumor-Intrinsic PD-L1 Promotes Breast Cancer Proliferation Through Livin and Galectin-1-Mediated Regulation of SKP2 Expression
Source: Int J Mol Sci. 2026 Mar 17;27(6):2741. doi: 10.3390/ijms27062741 (PMC13026925; doi:10.3390/ijms27062741)
Supplement: Supplementary file 1 [file ijms-27-02741-s001.zip › Supplementary Table 1.pdf]

Supplementary Table 1. SiRNA/ShRNA sequences

Target	Sequence

SiRNA sequences

	Sense	Antisense
PD-L1	GGC AUU UGC UGA ACG CAU UTT	AAU GCG UUC AGC AAA UGC CAG
PSMD2	UAU ACA UAG UGU GAU UUG Ctt	GCA AAU CAC ACU AUG UAU Att
Livin	GGA AGA GAC UUU GUC CAC Att	UGU GGA CAA AGU CUC UUC Ctt
EIF1AX	UUU AUU UAU GAU UAA AUC Ctt	GGA UUU AAU CAU AAA UAA Att
CALM2	UAA AUU UAA CAC AUU CUG Ctt	GCA GAA UGU GUU AAA UUU Att
TCF-3	UUA AUA CAA CGU UUA AUC Ctt	GGA UUA AAC GUU GUA UUA Att
Gal-1	UUU GAA UUC GUA UCC AUC Ctt	GGA UGG AUA CGA AUU CAA Att
SKP-2	UUA UAU AUG GAU AGU UUC Ctt	GGA AAC UAU CCA UAU AUA Att

ShRNA sequence

PD-L1	TGG TGC CGA CTA CAA GCG AAT TAC TGT GA
