# Supplementary material for: Tumor-Intrinsic PD-L1 Promotes Breast Cancer Proliferation Through Livin and Galectin-1-Mediated Regulation of SKP2 Expression
Source: Int J Mol Sci. 2026 Mar 17;27(6):2741. doi: 10.3390/ijms27062741 (PMC13026925; doi:10.3390/ijms27062741)
Supplement: Supplementary file 1 [file ijms-27-02741-s001.zip › Supplementary Table 2.pdf]

Supplementary Table 2. List of Antibodies used

Target	Clone	Cat#	Company, country
Livin	D61D1	5471	CST
Gal1	D608T	12936	CST
SKP2	D3G5	2652	CST
p27	D69C12	3686	CST
p21	12D1	2947	CST

CST= Cell Signaling Technology, Danvers, MA, USA, Gal1 = Galectin-1
