# Supplementary material for: Tumor-Intrinsic PD-L1 Promotes Breast Cancer Proliferation Through Livin and Galectin-1-Mediated Regulation of SKP2 Expression
Source: Int J Mol Sci. 2026 Mar 17;27(6):2741. doi: 10.3390/ijms27062741 (PMC13026925; doi:10.3390/ijms27062741)
Supplement: Supplementary file 1 [file ijms-27-02741-s001.zip › Supplementary Table 3.pdf]

Supplementary Table 3 Top 25 Differentially expressed proteins in the nuclear fraction of PD-L1 knockdown (PD-L1 (a)/(b) vs. PD-L1^{Pos} cells (Sh-CONT)

Numbers between brackets refer citations to references List in the "References" worksheet tab available in the "Supplementary' Dataset file (Excel)"

#	logFC	AveExpr	t	p-value	adj. P-value	B	Highest mean	Lowest mean	Gene /Protein Name	Known to affect cell Proliferation	Known to affect PI3K/AKT Pathway	Known to affect p21	Known to affect p27	Known to affect SKP2
1	-8.65	2.88	-55.06	0.000	0.000	17.01	**Sh-CONT**	Sh-PD-L1 (b)	SRRM2					
2	-8.07	3.34	-9.43	0.000	0.000	4.48	**Sh-CONT**	Sh-PD-L1 (a)	PSMD2	√ (1)	√ (2)	√ (1)	√ (1)	√ (1)
3	-7.91	3.78	-5.33	0.000	0.002	-0.18	**Sh-CONT**	Sh-PD-L1 (a)	BIRC7	√ (3)	√ (4)	√ (5)	√ (5)	
4	-7.44	4.29	-4.33	0.002	0.005	-1.66	**Sh-CONT**	Sh-PD-L1 (a)	PI4KA	√ (7,8)	√ (8)			
5	-7.34	3.20	-7.47	0.000	0.000	2.49	**Sh-CONT**	Sh-PD-L1 (a)	IGKV4-1					
6	-5.92	1.97	-43.69	0.000	0.000	16.03	**Sh-CONT**	Sh-PD-L1 (b)	ZNF862					
7	-5.33	9.73	-6.47	0.000	0.001	1.32	**Sh-CONT**	Sh-PD-L1 (b)	PROX2					
8	-5.23	5.40	-3.43	0.007	0.013	-3.08	**Sh-CONT**	Sh-PD-L1 (a)	PTPRF					
9	-5.04	10.13	-5.29	0.000	0.002	-0.23	**Sh-CONT**	Sh-PD-L1 (a)	RBMXL3					
10	-4.65	10.96	-11.20	0.000	0.000	5.98	**Sh-CONT**	Sh-PD-L1 (b)	MROH2A					
11	-4.59	8.38	-7.03	0.000	0.001	2.00	**Sh-CONT**	Sh-PD-L1 (a)	EIF1AX	√ (10,11)	√ (11)	√ (10)		
12	-4.56	2.20	-3.80	0.004	0.009	-2.48	**Sh-CONT**	Sh-PD-L1 (a)	SPG21	√ (6)				
13	-4.39	8.74	-4.52	0.001	0.005	-1.37	**Sh-CONT**	Sh-PD-L1 (a)	LMO7					
14	-4.31	9.02	-4.96	0.001	0.003	-0.71	**Sh-CONT**	Sh-PD-L1 (a)	TRPS1					
15	-4.27	8.57	-9.49	0.000	0.000	4.54	**Sh-CONT**	Sh-PD-L1 (a)	RPL21					
16	-4.21	5.44	-2.47	0.036	0.046	-4.68	**Sh-CONT**	Sh-PD-L1 (a)	ELOVL1	√ (16)				
17	-4.20	5.54	-2.74	0.023	0.031	-4.23	**Sh-CONT**	Sh-PD-L1 (b)	SNRPA	√ (9)				
18	-4.02	8.04	-5.37	0.000	0.002	-0.13	**Sh-CONT**	Sh-PD-L1 (a)	CABLES1					
19	-3.91	6.24	-4.29	0.002	0.005	-1.72	**Sh-CONT**	Sh-PD-L1 (a)	TCF3	√ (28,29)	√ (28)	√ (28)		
20	-3.51	11.30	-4.04	0.003	0.007	-2.11	**Sh-CONT**	Sh-PD-L1 (a)	CALM2	√ (13)	√ (13)	√ (14)	√ (15)	
21	-3.35	9.55	-5.75	0.000	0.002	0.40	**Sh-CONT**	Sh-PD-L1 (a)	LGALS1	√ (21)	√ (22)	√ (23)		
22	-3.26	8.61	-4.48	0.002	0.005	-1.43	**Sh-CONT**	Sh-PD-L1 (b)	CNOT1	√ (33)				
23	-3.02	4.05	-7.14	0.000	0.001	2.12	**Sh-CONT**	Sh-PD-L1 (a)	CALU	√ (32)				
24	-3.02	8.32	-3.59	0.006	0.011	-2.83	**Sh-CONT**	Sh-PD-L1 (b)	RAB35	√ (24)				
