# Supplementary figures and images for: Tumor-Intrinsic PD-L1 Promotes Breast Cancer Proliferation Through Livin and Galectin-1-Mediated Regulation of SKP2 Expression
Source: Int J Mol Sci. 2026 Mar 17;27(6):2741. doi: 10.3390/ijms27062741 (PMC13026925; doi:10.3390/ijms27062741)

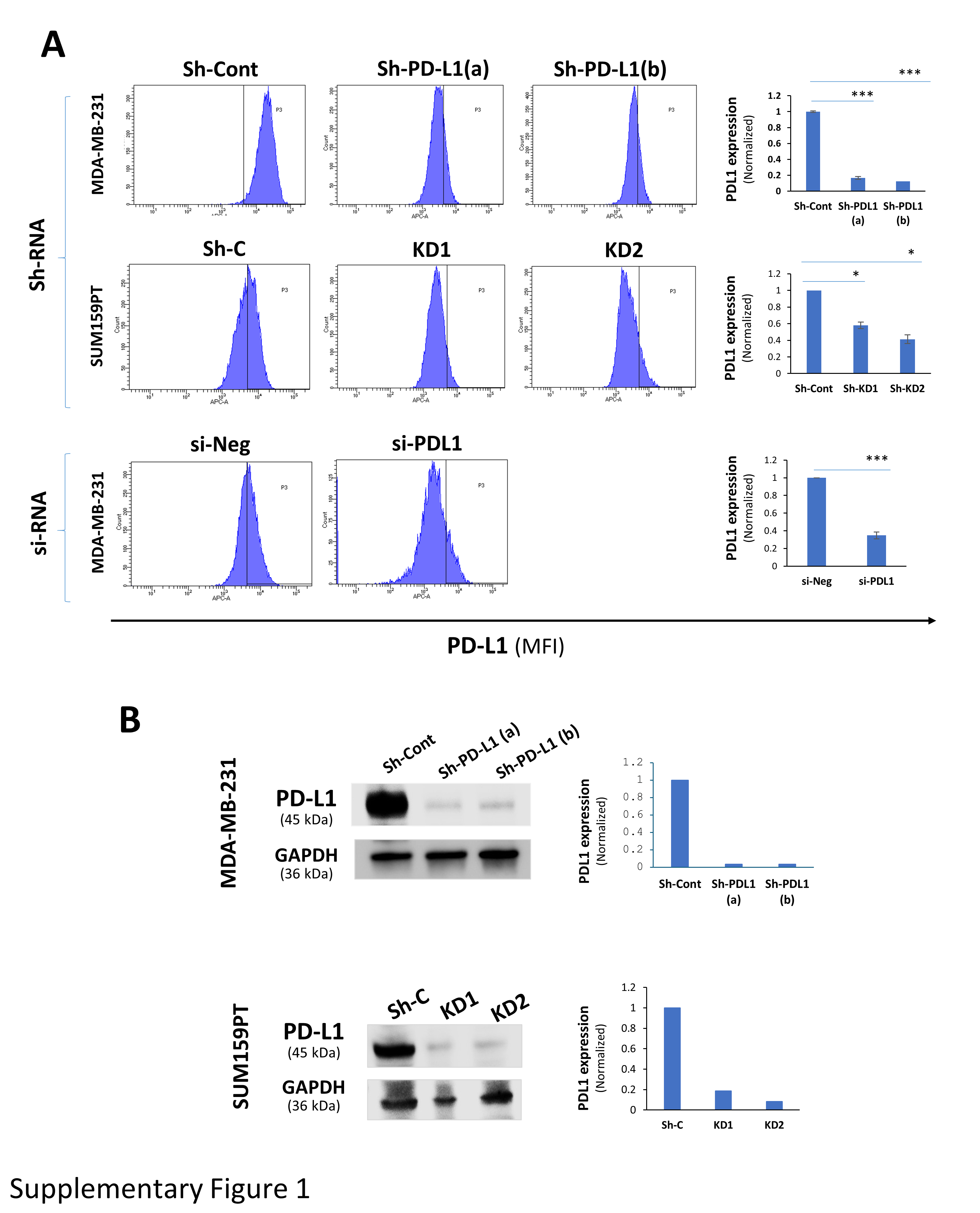

Supplement: Supplementary file 1 [file ijms-27-02741-s001.zip › Supplementary Figure 1.tif]

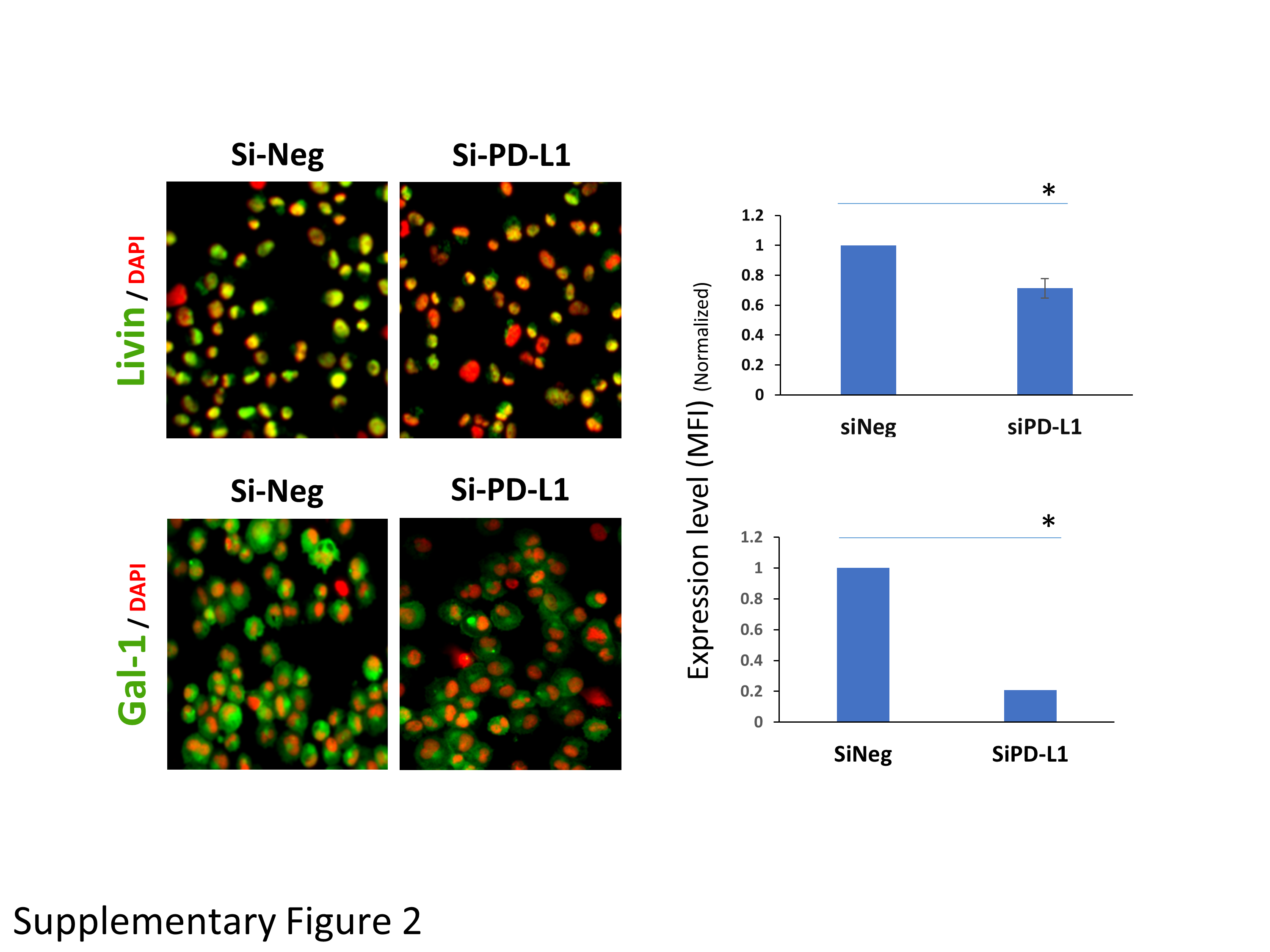

Supplement: Supplementary file 1 [file ijms-27-02741-s001.zip › Supplementary Figure 2.tif]

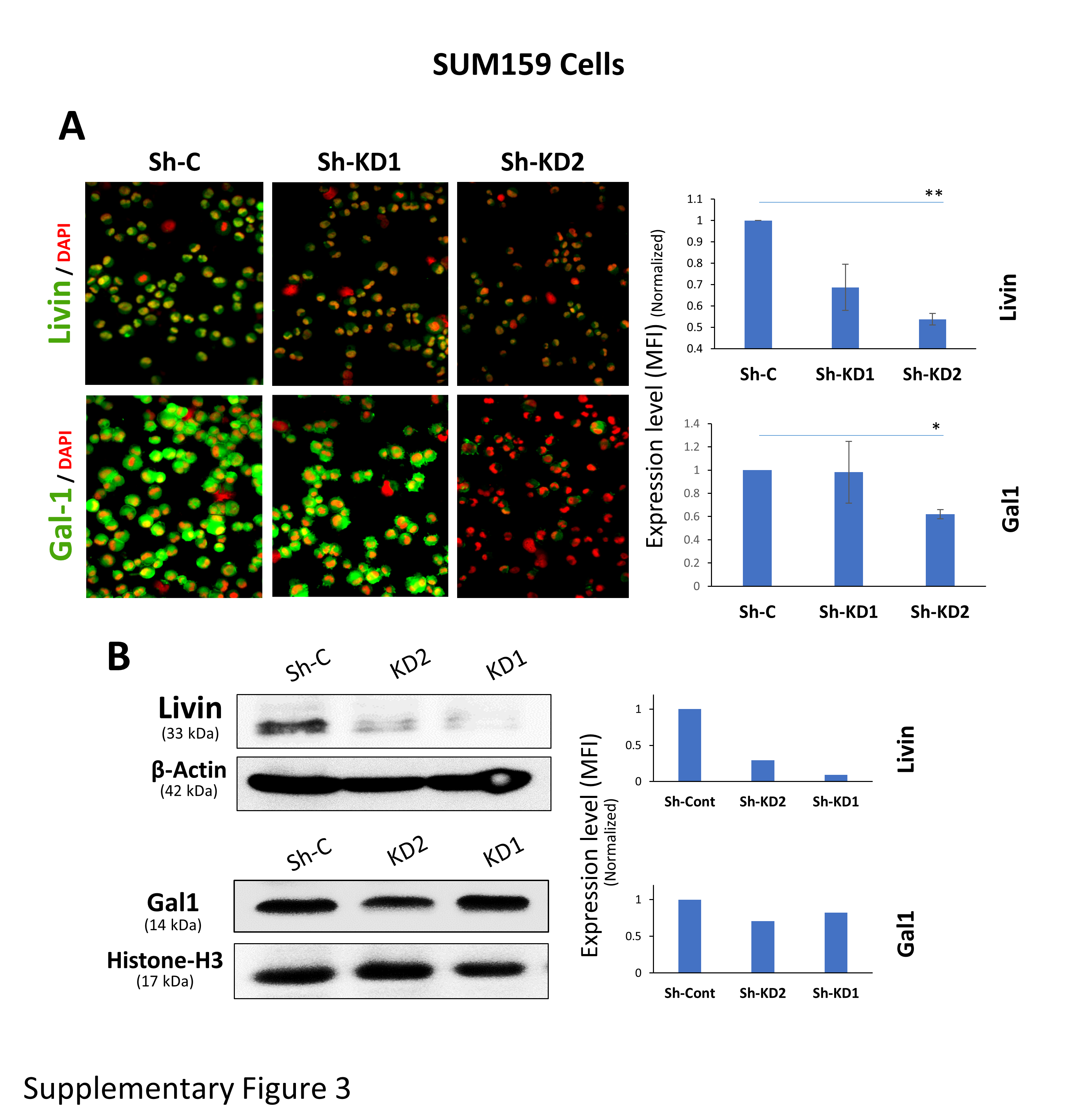

Supplement: Supplementary file 1 [file ijms-27-02741-s001.zip › Supplementary Figure 3.tif]

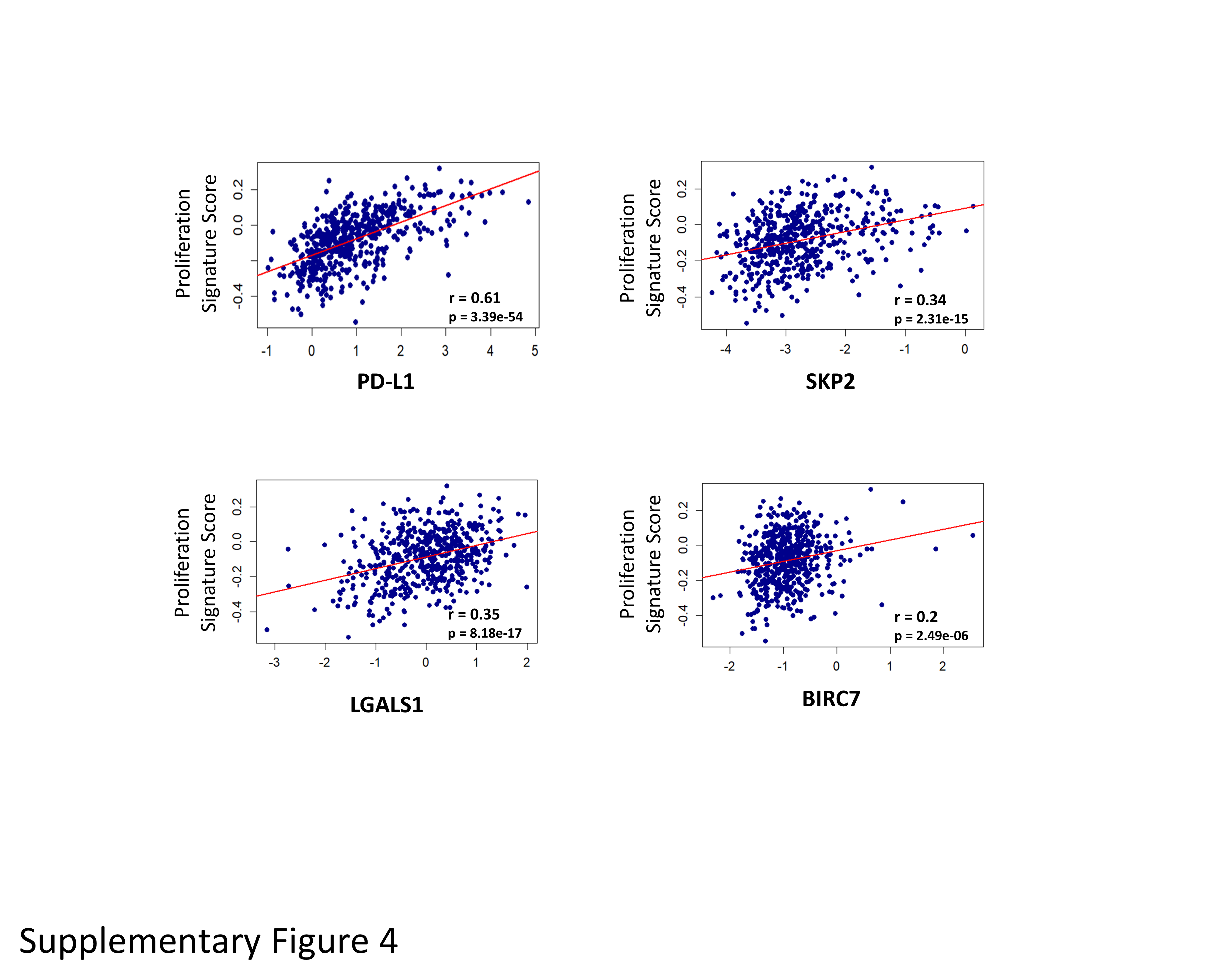

Supplement: Supplementary file 1 [file ijms-27-02741-s001.zip › Supplementary Figure 4.tif]
